# Local News

**Published:** 1885-07

**Authors:** 


### LOCAL NEWS.

PROFESSOR E. O. F. ROLER has been elected Emeritus Professor of Obstetrics in the Chicago Medical College. Adjunct-Professor W. W. Jaggard has been elected Professor of Obstetrics in the same college.

Dr. N. S. Davis, Jr., Lecturer on General Pathology and Pathological Anatomy, will supply, during the ensuing session, the vacancy caused by Professor Christian Fenger's resignation. Dr. Davis is about to return from a residence in Vienna and Heidelberg, where he has been engaged in the study of Pathology.

DR. SENN, of Milwaukee, Wisconsin, has accepted the Chair of Principles and Practice of Surgery in the College of Physicians and Surgeons, of Chicago, and Dr. Christian Fenger that of Clinical Surgery, in the same college.

SANITATION IN CHICAGO.—Dr. Oscar C. De Wolf, Commissioner of Health, of Chicago, has received an appropriation of $100,000 from the City Council for the purpose of improving the sanitary condition of Chicago. Thirty-four Sanitary Inspectors have been appointed, and it is intended that 50,000 houses shall be inspected before the first of September.

